# Conflicts of Interest Among Infectious Diseases Clinical Practice Guideline Authors and the Pharmaceutical Industry

**DOI:** 10.1001/jamanetworkopen.2023.8592

**Published:** 2023-04-17

**Authors:** Aileen S. Ahiskali, Dimitri M. Drekonja, Jonathan D. Alpern

**Affiliations:** 1 Department of Pharmacy, Hennepin Healthcare, Minneapolis, Minnesota; 2 Infectious Diseases Section, Minneapolis Veterans Affairs Healthcare System, Minneapolis, Minnesota; 3 Department of Medicine, University of Minnesota, Minneapolis; 4 Department of Infectious Diseases, HealthPartners, Minneapolis, Minnesota

## Abstract

This cross-sectional study assesses the prevalence of conflicts of interest (COI) associated with guideline-recommended drugs among Infectious Diseases Society of America clinical practice guideline authors and compliance with the Council on Medical Specialty Societies and Institute of Medicine guidelines.

## Introduction

Conflicts of interest (COI) among clinical practice guideline (CPG) authors are common^1,2,3^ and may affect clinician behavior, given the association that CPGs have with clinical decisions. The prevalence and nature of COI among authors of infectious diseases CPGs are poorly understood. We assessed the prevalence of COI associated with guideline-recommended drugs among Infectious Diseases Society of America (IDSA) CPG authors and compliance with Council on Medical Specialty Societies (CMSS) and Institute of Medicine (IOM) guidelines.^4,5^

## Methods

In this cross-sectional study, we searched all IDSA CPGs from June 1, 2017, to March 31, 2022 (N = 37). We excluded guidelines not published primarily by IDSA (n = 17) and guidelines without recommendations regarding specific antimicrobials (n = 10). We identified all CPG authors, their status as chair or co-chair, disclosed relationships, and relationship time frames. We included relationships only with pharmaceutical companies that developed or sold drugs. We extracted all drugs recommended in each CPG and excluded drugs recommended only in a clinical trial or sold only outside the US. We searched US Food and Drug Administration websites to identify drug manufacturers and approval dates. This study followed the Strengthening the Reporting of Observational Studies in Epidemiology (STROBE) reporting guideline and was determined to be exempt by the HealthPartners institutional review board because it was non–human participants research.

Disclosed relationships were categorized as advisory, industry-sponsored research, stockholder, royalties, honoraria, or other. We defined COI as any disclosed relationship between a CPG author (or spouse) and a pharmaceutical company if the company manufactured a guideline-recommended drug at the time of publication. Companies associated with a manufacturer as a subsidiary or acquisition were included. Conflicts of interest described in the present tense were considered active relationships and categorized as high-level COI (eMethods, eFigure, and eTable in Supplement 1).

We assessed the prevalence of COI and high-level COI and guideline compliance with the following IOM and CMSS recommendations: (1) most panel members are free of COI relevant to guideline subject matter and (2) panel chairs (or ≥1 chair if co-chairs) are free of COI relevant to guideline subject matter.^4,5^ We used Microsoft Excel, version 2301 (Microsoft Corp), for the analysis.

## Results

Among 10 IDSA CPGs, approximately one-half of authors (71 of 149 [47.7%]) disclosed a relationship with any pharmaceutical company, and one-third (48 of 149 [32.2%]) had 1 or more COI or high-level COI (Table 1). The antimicrobial resistance (AMR 2022) CPG had the most COI (23), followed by nontuberculous mycobacteria (NTM 2020) (22), *Clostridioides difficile* infection (CDI 2018) (21), and COVID-19 2021 (12) (Table 2). Most CPGs (9 [90.0%]) had authors with at least 1 COI (Table 1). Four CPGs were nonadherent to IOM and CMSS standards because at least one-half of authors had COI (AMR 2022, CDI 2018, CDI 2021, and NTM 2020) and 1 CPG was nonadherent because of chairs having relevant COI (CDI 2021).

**Table 1.** Industry Relationship and COI Summary

IDSA guideline	No. (%)										
	Overall^a^	Antimicrobial resistance 2022	Babesiosis 2020	*Clostridium difficile* 2018	*Clostridioides difficile* 2021	COVID-19 2021	Infectious diarrhea 2017	Influenza 2018	Lyme 2020	Neurocysticercosis 2018	Nontuberculous mycobacteria 2020
Total authors	149	6	14	14	7	16	12	18	32	9	21
Authors disclosing any industry relationship, No. (%)	71 (47.7)	5 (83.3)	5 (35.7)	7 (50.0)	4 (57.1)	5 (31.3)	5 (41.7)	9 (50.0)	20 (62.5)	0	11 (52.4)
Authors with COI, No. (%)	48 (32.2)	5 (83.3)	2 (14.3)	7 (50.0)	4 (57.1)	5 (31.3)	2 (16.7)	5 (27.8)	7 (21.9)	0	11 (52.4)
Majority of panel members are free of relevant COI, No./total No. (%)	6/10 (60.0)	No	Yes	No	No	Yes	Yes	Yes	Yes	Yes	No
Panel chair(s) is/are free of relevant COI, No./total No. (%)	9/10 (90.0)	Yes^b^	Yes	Yes^b^	No	Yes^b^	Yes^b^	Yes^b^	Yes	Yes^b^	Yes

Abbreviations: Antimicrobial Resistance 2022, IDSA Antimicrobial Resistant Treatment Guidance: Gram-Negative Bacterial Infections; Babesiosis 2020, Clinical Practice Guidelines by IDSA: 2020 Guideline on Diagnosis and Management of Babesiosis; *Clostridioides difficile* 2021, Clinical Practice Guideline by IDSA and SHEA: 2021 Focused Update Guidelines on Management of *Clostridioides difficile* Infection in Adults; *Clostridium difficile* 2018, Clinical Practice Guidelines for *Clostridium difficile* Infection in Adults and Children: 2017 Update by IDSA and SHEA; COI, conflicts of interest; COVID-19 2021, IDSA Guidelines on the Treatment and Management of Patients with COVID-19; IDSA, Infectious Diseases Society of America; Infectious diarrhea 2017, 2017 IDSA Clinical Practice Guidelines for the Diagnosis and Management of Infectious Diarrhea; Influenza 2018, Clinical Practice Guidelines by IDSA: 2018 Update on Diagnosis, Treatment, Chemoprophylaxis, and Institutional Outbreak Management of Seasonal Influenza; Lyme 2020, Clinical Practice Guidelines by IDSA, AAN, and ACR: 2020 Guidelines for the Prevention, Diagnosis and Treatment of Lyme Disease; Neurocysticercosis 2018, Diagnosis and Treatment of Neurocysticercosis: 2017 Clinical Practice Guidelines by IDSA and ASTMH; Nontuberculous mycobacteria 2020, Treatment of Nontuberculous Mycobacterial Pulmonary Disease: An Official ATS/ERS/ESCMID/IDSA Clinical Practice Guideline.

^a^ Overall assessment is based on 10 guidelines.

^b^ Corresponding authors listed but no chair or co-chair listed.

**Table 2.** Characteristics of COI Between Infectious Diseases Society of America Clinical Practice Guideline Authors and the Pharmaceutical Industry

Relationship	Overall^a^	Antimicrobial resistance 2022	Babesiosis 2020	*Clostridium difficile* 2018	*Clostridioides difficile* 2021	COVID-19 2021	Infectious diarrhea 2017	Influenza 2018	Lyme 2020	Neurocysticercosis 2018	Nontuberculous mycobacteria 2020
**Overall COI**											
Total COI^b^	104	23	2	21	7	12	2	7	8	0	22
High-level COI	23	10	0	0	2	9	0	2	0	0	0
**Advisory**											
Total COI^b^	50	12	1	10	4	6	1	3	3	0	10
High-level COI	10	5	0	0	1	4	0	0	0	0	0
**Honoraria**											
Total COI^b^	4	2	0	1	1	0	0	0	0	0	0
High-level COI	2	1	0	0	1	0	0	0	0	0	0
**Industry-sponsored research**											
Total COI^b^	34	8	0	5	2	5	1	1	4	0	8
High-level COI	9	4	0	0	0	5	0	0	0	0	0
**Other**											
Total COI^b^	7	1	0	0	0	1	0	0	1	0	4
High-level COI	0	0	0	0	0	0	0	0	0	0	0
**Royalties**											
Total COI^b^	8	0	0	5	0	0	0	3	0	0	0
High-level COI	2	0	0	0	0	0	0	2	0	0	0
**Stockholder**											
Total COI^b^	1	0	1	0	0	0	0	0	0	0	0
High-level COI	0	0	0	0	0	0	0	0	0	0	0

Abbreviations: Antimicrobial Resistance 2022, IDSA Antimicrobial Resistant Treatment Guidance: Gram-Negative Bacterial Infections; Babesiosis 2020, -Clinical Practice Guidelines by IDSA: 2020 Guideline on Diagnosis and Management of Babesiosis; *Clostridioides difficile* 2021, Clinical Practice Guideline by IDSA and SHEA: 2021 Focused Update Guidelines on Management of *Clostridioides difficile* Infection in Adults; *Clostridium difficile* 2018, Clinical Practice Guidelines for *Clostridium difficile* Infection in Adults and Children: 2017 Update by IDSA and SHEA; COI, conflicts of interest; COVID-19 2021, IDSA Guidelines on the Treatment and Management of Patients with COVID-19; IDSA, Infectious Diseases Society of America; Infectious diarrhea 2017, 2017 IDSA Clinical Practice Guidelines for the Diagnosis and Management of Infectious Diarrhea; Influenza 2018, Clinical Practice Guidelines by IDSA: 2018 Update on Diagnosis, Treatment, Chemoprophylaxis, and Institutional Outbreak Management of Seasonal Influenza; Lyme 2020, Clinical Practice Guidelines by IDSA, AAN, and ACR: 2020 Guidelines for the Prevention, Diagnosis and Treatment of Lyme Disease; Neurocysticercosis 2018, Diagnosis and Treatment of Neurocysticercosis: 2017 Clinical Practice Guidelines by IDSA and ASTMH; Nontuberculous Mycobacteria 2020, Treatment of Nontuberculous Mycobacterial Pulmonary Disease: An Official ATS/ERS/ESCMID/IDSA Clinical Practice Guideline.

^a^ Authors may be counted more than once if they have multiple types of COI and/or COI with different companies.

^b^ Total COI includes COI plus high-level COI.

## Discussion

Relationships between IDSA CPG authors and the pharmaceutical industry are common. Approximately one-third of authors disclosed relationships with manufacturers of guideline-recommended drugs, and multiple IDSA CPGs were nonadherent to CMSS and IOM standards. Our findings warrant further attention considering that even the appearance of bias in CPGs can have negative consequences—public distrust in science and health care, including concerns about ties between clinicians and pharmaceutical companies, has been increasing. Study limitations are that we did not assess the accuracy of COI disclosures using third-party data, which may have resulted in underreporting of COI.^6^ We also focused on the tense of disclosures in our analysis, which is vulnerable to misclassification. To ensure that IDSA CPGs remain a trusted source, improving the review and management process for CPG COI is needed.

## Notes

Supplement 1.
eMethods.

eReferences.
**eTable.** IDSA Guideline Assessment **eFigure.** IDSA Guideline Breakdown and Citations of Included Guidelines

Supplement 2.
Data Sharing Statement
