# Supplementary material for: Conflicts of Interest Among Infectious Diseases Clinical Practice Guideline Authors and the Pharmaceutical Industry
Source: JAMA Netw Open. 2023 Apr 17;6(4):e238592. doi: 10.1001/jamanetworkopen.2023.8592 (PMC10111177; doi:10.1001/jamanetworkopen.2023.8592)
Supplement: Supplement 1. — eMethods. eReferences. eTable. IDSA Guideline Assessment eFigure. IDSA Guideline Breakdown and Citations of Included Guidelines [file jamanetwopen-e238592-s001.pdf]

Supplementary Online Content

Ahiskali AS, Drekonja DM, Alpern JD. Conflicts of interest among infectious diseases clinical practice guideline authors and the pharmaceutical industry. *JAMA Netw Open.* 2023;6(4):e238592. doi:10.1001/jamanetworkopen.2023.8592

eMethods.
eReferences.
eTable. IDSA Guideline Assessment
eFigure. IDSA Guideline Breakdown and Citations of Included Guidelines

This supplementary material has been provided by the authors to give readers additional information about their work.

© 2023 Ahiskali AS et al. *JAMA Network Open.*

eMethods.

Data sources:

June 2017 was chosen as the start date for data collection of the IDSA CPGs because this was when IDSA reported adopting the CMSS Code of Interactions with Companies.[1] We excluded guidelines that were not published primarily by IDSA, as it was not possible to ensure that the same methods for assessing COIs were utilized by each external organization.

Data extraction:

Of the CPGs meeting inclusion criteria, we only included relationships with pharmaceutical companies that sold or developed drugs. We excluded relationships with companies that only sold vaccines or diagnostic products because specific brands were not consistently specified and thus COIs could not be reasonably adjudicated. We also excluded relationships with companies that could not be identified using multiple internet/database searches, were bankrupt prior to the publication of the CPG, and governmental organizations. For one CPG (Lyme 2020), patient and consumer representatives were included as authors (N = 4), however the three patient representatives were listed anonymously. We chose to exclude these authors from the analysis as it was unclear what involvement they had in the development of treatment recommendations.

For the extraction of drug products in the IDSA guidelines, we did not consider drugs in which a drug class, but not a specific drug product, was recommended (i.e., "aminoglycosides"). If a drug's specific formulation was listed in a CPG, we only considered manufacturers of that formulation(s). We searched the Food and Drug Administration's (FDA) Orange Book (generic and brand-name drugs), Purple Book (biologic drugs), and other FDA websites (investigational drugs) to identify each drug's manufacturer(s) and the dates of FDA approval.

Outcomes:

We categorized each author's disclosed relationships as advisory, industry-sponsored research, stockholder, royalties, honoraria, or other (defined as travel and speaking-related expenses) and determined whether there was a COI or high-level COI present. The IDSA defines a potential COI as a "panel member…with financial or other interest

© 2023 Ahiskali AS et al. *JAMA Network Open.*

that might bias his or her decisions or actions concerning matters before the guideline panel."[1] Most studies use a low threshold for defining COIs (e.g., any disclosed relationship, regardless of the company's relevance to a particular guideline). In order to avoid overstating the significance of a disclosed relationship, we chose to use a strict definition of a COI. We defined a COI as any disclosed relationship, past or present, between a CPG author (or spouse) and a pharmaceutical company, if the company was an active manufacturer of a drug recommended in that guideline at the time of publication. Additionally, we considered a COI to be any relationship between an author and a company associated with a guideline manufacturer as a subsidiary or acquisition, which was determined using multiple sources, including Crunchbase database,[2] company websites, and Google searches. Each COI was independently reviewed and categorized by two of the study authors (ASA and JDA), and a third author (DMD) was used to establish consensus.

We considered COIs described in the present tense (e.g., receiv*es* honoraria) to be an active relationship and categorized these as high-level COIs as defined by the American College of Physicians Clinical Guidelines Committee.[3] The exception to this rule was for active relationships involving a generic drug with multiple manufacturers. We defined these relationships as COIs, instead of high-level COIs, because the presence of multiple manufacturers likely decreases the risk of bias in these scenarios. In circumstances where it was not possible to assess the timeframe of a relationship (i.e., lacked clarity regarding whether it was a present or past relationship), we downgraded our assessment of the COI from high-level COI to COI.

© 2023 Ahiskali AS et al. *JAMA Network Open*.

eReferences.

1. Clinical Practice Guidelines Development: Training and Resources. https://www.idsociety.org/practice-guideline/clinical-practice-guidelines-development-training-and-resources/. Accessed June 26, 2022.
2. Cruchbase Database. https://www.crunchbase.com/. Accessed May 20, 2022.
3. Qaseem A, Wilt TJ, Forciea MA, et al. Disclosure of interests and management of conflicts of interest in clinical guidelines and guidance statements: methods from the clinical guidelines committee of the American College of Physicians. *Ann Intern Med.* 2019;171:354-61.

© 2023 Ahiskali AS et al. *JAMA Network Open.*

eTable. IDSA Guideline Assessment

Date	**Guideline**<br>***Journal/Website of Publication***	Included	Reason(s) for Exclusion
09/2017	HCV Guidance: Recommendations for Testing, Managing, and Treating Hepatitis C<br>*HCVGuidelines.org*	No	Published in non-IDSA journal/platform
09/2017	2017 HRS expert consensus statement on cardiovascular implantable electronic device lead management and extraction<br>*Heart Rhythm Society*	No	Published in non-IDSA journal/platform
09/2017	2017 HIVMA of IDSA Clinical Practice Guideline for the Management of Chronic Pain in Patients Living With HIV<br>*Clinical Infectious Diseases*	No	No antimicrobial therapy recommendations
10/2017	2017 Infectious Diseases Society of America Clinical Practice Guidelines for the Diagnosis and Management of Infectious Diarrhea **(DIA 2017)**<br>*Clinical Infectious Diseases*	Yes	N/A
11/2017	Outbreak Response and Incident Management: SHEA Guidance and Resources for Healthcare Epidemiologists in United States Acute-Care Hospitals<br>*Infection Control & Hospital Epidemiology*	No	Published in non-IDSA journal/platform
02/2018	Clinical Practice Guidelines for Clostridium difficile Infection in Adults and Children: 2017 Update by the Infectious Diseases Society of America (IDSA) and Society for Healthcare Epidemiology of America (SHEA) **(CDI 2018)**<br>*Clinical Infectious Diseases*	Yes	N/A
02/2018	Diagnosis and Treatment of Neurocysticercosis: 2017 Clinical Practice Guidelines by the Infectious Diseases Society of America (IDSA) and the American Society of Tropical Medicine and Hygiene (ASTMH) **(NCC 2018)**<br>*Clinical Infectious Diseases*	Yes	N/A
05/2018	Outpatient Management of Fever and Neutropenia in Adults Treated for Malignancy: American Society of Clinical Oncology and Infectious Diseases Society of America Clinical Practice Guideline Update<br>*Journal of Clinical Oncology*	No	Published in non-IDSA journal/platform
06/2018	A Guide to Utilization of the Microbiology Laboratory for Diagnosis of Infectious Diseases: 2018 Update by the Infectious Diseases Society of America and the American Society for Microbiology<br>*Clinical Infectious Diseases*	No	No antimicrobial therapy recommendations
08/2018	SHEA neonatal intensive care unit (NICU) white paper series: Practical approaches to Clostridioides difficile prevention<br>*Infection Control & Hospital Epidemiology*	No	No antimicrobial therapy recommendations

© 2023 Ahiskali AS et al. *JAMA Network Open.*

Date	**Guideline**<br>***Journal/Website of Publication***	Included	Reason(s) for Exclusion
09/2018	Antimicrobial Prophylaxis for Adult Patients with Cancer Related Immunosuppression: ASCO and IDSA Clinical Practice Guideline Update<br>*Journal of Clinical Oncology*	No	Published in non-IDSA journal/platform
11/2018	2018 Infectious Diseases Society of America Clinical Practice Guideline for the Management of Outpatient Parenteral Antimicrobial Therapy<br>*Clinical Infectious Diseases*	No	No antimicrobial therapy recommendations
12/2018	Clinical Practice Guidelines by the Infectious Diseases Society of America: 2018 Update on Diagnosis, Treatment, Chemoprophylaxis, and Institutional Outbreak Management of Seasonal Influenza **(FLU 2018)**<br>*Clinical Infectious Diseases*	Yes	N/A
02/2019	Clinical Practice Guideline: Tonsillectomy in Children (Update)<br>*Otolaryngology - Head and Neck Surgery*	No	Published in non-IDSA journal/platform
03/2019	Diagnosis & Prevention of Periprosthetic Joint Infections<br>*American Academy of Orthopaedic Surgeons website*	No	Published in non-IDSA journal/platform
03/2019	Clinical Practice Guideline for the Management of Asymptomatic Bacteriuria: 2019 Update by the Infectious Diseases Society of America<br>*Clinical Infectious Diseases*	No	No antimicrobial therapy recommendations
10/2019	Diagnosis and Treatment of Adults with Community-acquired Pneumonia An Official Clinical Practice Guideline of the American Thoracic Society and Infectious Diseases Society of America<br>*American Thoracic Society Documents*	No	Published in non-IDSA journal/platform
11/2019	Treatment of Drug-Resistant Tuberculosis. An Official ATS/CDC/ERS/IDSA Clinical Practice Guideline<br>*American Journal of Respiratory and Critical Care Medicine*	No	Published in non-IDSA journal/platform
12/2019	Guidelines for the Prevention and Treatment of Opportunistic Infections in HIV-Exposed and HIV-Infected Children<br>*AIDSinfo.gov (renamed to clinicalinfo.hiv.gov)*	No	Published in non-IDSA journal/platform
03/2020	Therapeutic monitoring of vancomycin for serious methicillin-resistant Staphylococcus aureus infections: A revised consensus guideline and review by the American Society of Health-System Pharmacists, the Infectious Diseases Society of America, the Pediatric Infectious Diseases Society, and the Society of Infectious Diseases Pharmacists<br>*American Journal of Health System Pharmacy*	No	Published in non-IDSA journal/platform

© 2023 Ahiskali AS et al. *JAMA Network Open.*

Date	**Guideline**<br>***Journal/Website of Publication***	Included	Reason(s) for Exclusion
07/2020	Treatment of Nontuberculous Mycobacterial Pulmonary Disease: An Official ATS/ERS/ESCMID/IDSA Clinical Practice Guideline **(NTM 2020)**<br>*Clinical Infectious Diseases*	Yes	N/A
08/2020	Guidelines for the Prevention and Treatment of Opportunistic Infections in Adults and Adolescents with HIV<br>*AIDSinfo.gov (renamed to clinicalinfo.hiv.gov)*	No	Published in non-IDSA journal/platform
08/2020	Infectious Diseases Society of America Guidelines on the Diagnosis of COVID-19: Serologic Testing<br>*IDSA Website*	No	No antimicrobial therapy recommendations
09/2020	SHEA neonatal intensive care unit (NICU) white paper series: Practical approaches to Staphylococcus aureus disease prevention<br>*Infection Control & Hospital Epidemiology*	No	Published in non-IDSA journal/platform
10/2020	Management of healthcare personnel living with hepatitis B, hepatitis C, or human immunodeficiency virus in US healthcare institutions<br>*Infection Control & Hospital Epidemiology*	No	Published in non-IDSA journal/platform
11/2020	Clinical Practice Guidelines by the Infectious Diseases Society of America (IDSA): 2020 Guideline on Diagnosis and Management of Babesiosis **(BAB 2020)**<br>*Clinical Infectious Diseases*	Yes	N/A
11/2020	Clinical Practice Guidelines by the Infectious Diseases Society of America (IDSA), American Academy of Neurology (AAN), and American College of Rheumatology (ACR): 2020 Guidelines for the Prevention, Diagnosis and Treatment of Lyme Disease **(Lyme 2020)**<br>*Clinical Infectious Diseases*	Yes	N/A
12/2020	Reliability of nonlocalizing signs and symptoms as indicators of the presence of infection in nursing-home residents<br>*Infection Control & Hospital Epidemiology*	No	Published in non-IDSA journal/platform
12/2020	IDSA Guidelines on the Diagnosis of COVID-19: Molecular Diagnostic Testing<br>*IDSA Website*	No	No antimicrobial therapy recommendations
05/2021	The Infectious Diseases Society of America Guidelines on the Diagnosis of COVID-19: Antigen Testing<br>*IDSA Website*	No	No antimicrobial therapy recommendations

© 2023 Ahiskali AS et al. *JAMA Network Open.*

Date	**Guideline**<br>***Journal/Website of Publication***	Included	Reason(s) for Exclusion
06/2021	Clinical Practice Guideline by the Infectious Diseases Society of America (IDSA) and Society for Healthcare Epidemiology of America (SHEA): 2021 Focused Update Guidelines on Management of Clostridioides difficile Infection in Adults **(CDI 2021)**<br>*Clinical Infectious Diseases*	Yes	N/A
07/2021	Early Care of Adults with Suspected Sepsis in the Emergency Department and Out-of-Hospital Environment: A Consensus-Based Task Force Report<br>*Annals of Emergency Medicine*	No	Published in non-IDSA journal/platform
08/2021	Clinical Practice Guideline by the Pediatric Infectious Diseases Society and the Infectious Diseases Society of America: 2021 Guideline on Diagnosis and Management of Acute Hematogenous Osteomyelitis in Pediatrics<br>*Journal of the Pediatric Infectious Diseases Society*	No	Published in non-IDSA journal/platform
10/2021	IDSA Guidelines on the Treatment and Management of Patients with COVID-19 **(COVID 2021)**<br>*IDSA Website*	Yes	N/A
11/2021	Infectious Diseases Society of America Guidelines on Infection Prevention for Health Care Personnel Caring for Patients with Suspected or Known COVID-19<br>*IDSA Website*	No	No antimicrobial therapy recommendations
12/2021	Primary Care Guidance for Persons with Human Immunodeficiency Virus: 2020 Update by the HIV Medicine Association of the Infectious Diseases Society of America<br>*Clinical Infectious Diseases*	No	No antimicrobial therapy recommendations
12/2021	Surviving Sepsis Campaign: International Guidelines for Management of Sepsis and Septic Shock 2021<br>*Intensive Care Medicine*	No	Published in non-IDSA journal/platform
03/2022	Infectious Diseases Society of America 2022 Guidance on the Treatment of Extended-Spectrum β-lactamase Producing Enterobacterales, Carbapenem-Resistant Enterobacterales, and Pseudomonas aeruginosa with Difficult-to-Treat Resistance (Version 1.1) **and** Guidance on the Treatment of AmpC β-lactamase-Producing Enterobacterales, Carbapenem-resistant Acinetobacter baumanii, and Stenotrophomonas maltophilia Infections (Version 2.0) **(AMR 2022)**<br>*IDSA Website*	Yes	N/A

© 2023 Ahiskali AS et al. *JAMA Network Open.*

eFigure. IDSA Guideline Breakdown and Citations of Included Guidelines

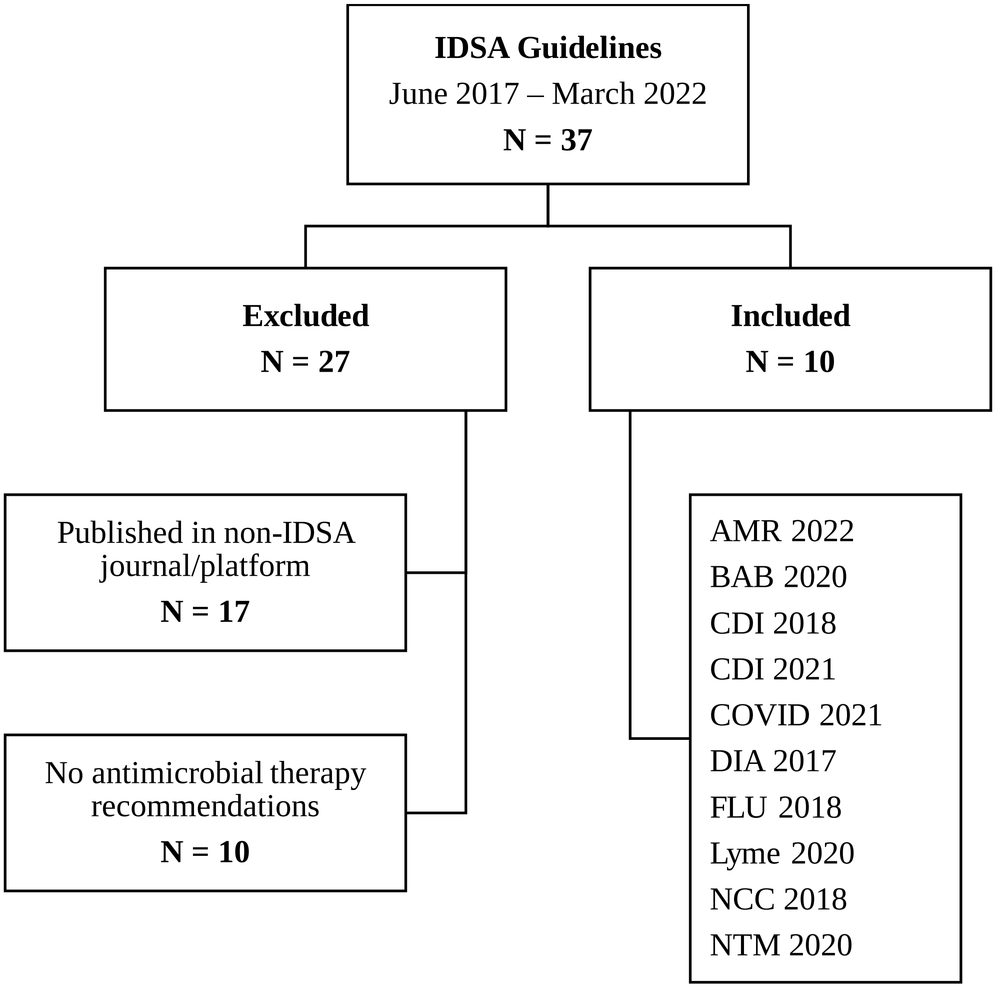

<u>**Abbreviations & citations of included guidelines**</u>

- **AMR 2022** = Antimicrobial resistance
  Tamma PD, Aitken SL, Bonomo RA, Mathers AJ, van Duin D, Clancy CJ. Infectious Diseases Society of America antimicrobial-resistant treatment guidance: gram-negative bacterial infections. Infectious Diseases Society of America 2022; Version 1.0. Available at https://www.idsociety.org/practice-guideline/amr-guidance/. Accessed June 25, 2022.
- **BAB 2020** = Babesiosis
  Krause PJ, Auwaerter PG, Bannuru RR, et al. Clinical practice guidelines by the Infectious Diseases Society of America (IDSA): 2020 guideline on diagnosis and management of babesiosis. *Clin Infect Dis*. 2021;72:e49-e64.
- **CDI 2018** = *Clostridium difficile*
  McDonald LC, Gerding DN, Johnson S, et al. Clinical practice guidelines for *Clostridium difficile* infection in adults and children: 2017 update by the Infectious Diseases Society of America (IDSA) and Society for Healthcare Epidemiology of America (SHEA). *Clin Infect Dis*. 2018;66:e1-e48.
- **CDI 2021** = *Clostridium difficile*
  Johnson S, Lavergne V, Skinner AM, et al. Clinical practice guideline by the Infectious Diseases Society of America (IDSA) and Society for Healthcare Epidemiology of America (SHEA): 2021 focused update guidelines of management of *Clostridium difficile* infection in adults.
- **COVID 2021** = COVID-19
  Bhimraj A, Morgan RL, Hirsch Shumaker A, et al. Infectious Diseases Society of America guidelines on the treatment and management of patients with COVID-19. Infectious Diseases Society of America 2022; version 9.0.0. Available at: https://www.idsociety.org/practice-guideline/covid-19-guideline-treatment-and-management/. Accessed June 27, 2022.
- **DIA 2017** = Infectious Diarrhea

© 2023 Ahiskali AS et al. *JAMA Network Open.*

Shane AL, Mody RK, Crump JA, et al. 2017 Infectious Diseases Society of America clinical practice guidelines for the diagnosis and management of infectious diarrhea. *Clin Infect Dis*. 2017;65:e45-e80.

- **FLU 2018** = Influenza
  Uyeki TM, Bernstein HH, Bradley JS, et al. Clinical practice guidelines by the Infectious Diseases Society of America: 2018 update on diagnosis, treatment, chemoprophylaxis, and institutional outbreak management of seasonal influenza. *Clin Infect Dis*. 2019;68:e1-e47.
- **Lyme 2020** = Lyme Disease
  Lantos PM, Rumbaugh J, Bockenstedt LK, et al. Clinical practice guidelines by the Infectious Diseases Society of America (IDSA), American Academy of Neurology (AAN), and American College of Rheumatology (ACR): 2020 guidelines for the prevention, diagnosis and treatment of Lyme disease. *Clin Infect Dis*. 2021;71:e1-48.
- **NCC 2018** = Neurocysticercosis
  White AC, Coyle CM, Rajshekhar, et al. Diagnosis and treatment of neurocysticercosis: 2017 clinical practice guidelines by the Infectious Diseases Society of America (IDSA) and the American Society of Tropical Medicine and Hygiene (ASTMH). *Clin Infect Dis*. 2018;66:e49-e75.
- **NTM 2020** = Nontuberculous mycobacterial infections
  Daley CL, Iaccarino JM, Lange C, et al. Treatment of nontuberculous mycobacterial pulmonary disease: an official ATS/ERS/ESCMID/IDSA clinical practice guideline. *Clin Infect Dis*. 2020;71:e1-e36.

© 2023 Ahiskali AS et al. *JAMA Network Open.*
