# Supplementary material for: Conflicts of Interest Among Infectious Diseases Clinical Practice Guideline Authors and the Pharmaceutical Industry
Source: JAMA Netw Open. 2023 Apr 17;6(4):e238592. doi: 10.1001/jamanetworkopen.2023.8592 (PMC10111177; doi:10.1001/jamanetworkopen.2023.8592)
Supplement: Supplement 2. — Data Sharing Statement [file jamanetwopen-e238592-s002.pdf]

Data Sharing Statement

Ahiskali. Conflicts of Interest Among Infectious Diseases Clinical Practice Guideline Authors and the Pharmaceutical Industry. *JAMA Netw Open*. Published April 17, 2023. doi:10.1001/jamanetworkopen.2023.8592

Data
Data available: No

Additional Information
Explanation for why data not available: We would be happy to make our dataset available to the Editor and peer reviewers upon request. While all data are publicly available, we do not intend to emphasize individual authors' conflicts of interest. If the editorial team recommends including our dataset in the final publication, we would be willing to oblige this request.
